# Unmasking the Invisible Threat: Biological Impacts and Mechanisms of Polystyrene Nanoplastics on Cells

**DOI:** 10.3390/toxics12120908

**Published:** 2024-12-14

**Authors:** Wenxia Bu, Ye Cui, Yueyuan Jin, Xuehai Wang, Mengna Jiang, Ruiyao Huang, JohnPaul Otuomasiri Egbobe, Xinyuan Zhao, Juan Tang

**Affiliations:** 1 Nantong Key Laboratory of Environmental Toxicology, Department of Occupational Medicine and Environmental Toxicology, School of Public Health, Nantong University, Nantong 226019, China; buwenxia02@163.com (W.B.); cuiye2839@163.com (Y.C.); 2417310031@stmail.ntu.edu.cn (Y.J.); lankeqing3910@gmail.com (X.W.); 17851790126@163.com (M.J.); johnegbobepaul@gmail.com (J.O.E.); 2 Department of Clinical Medicine, Nantong University Xinglin College, Nantong 226000, China; hry20041014@163.com

**Keywords:** polystyrene nanoplastics, cellular toxicity, oxidative stress, autophagy, cell death

## Abstract

Polystyrene nanoplastics (PS-NPs), a pervasive component of plastic pollution, have emerged as a significant environmental and health threat due to their microscopic size and bioaccumulative properties. This review systematically explores the biological effects and mechanisms of PS-NPs on cellular systems, encompassing oxidative stress, mitochondrial dysfunction, DNA damage, inflammation, and disruptions in autophagy. Notably, PS-NPs induce multiple forms of cell death, including apoptosis, ferroptosis, necroptosis, and pyroptosis, mediated through distinct yet interconnected molecular pathways. The review also highlights various factors that influence the cytotoxicity of PS-NPs, such as particle size, surface modifications, co-exposure with other pollutants, and protein corona formation. These complex interactions underscore the extensive and potentially hazardous impacts of PS-NPs on cellular health. The findings presented here emphasize the need for continued research on the mechanisms underlying PS-NP toxicity and the development of effective strategies for mitigating their effects, thereby informing regulatory frameworks aimed at minimizing environmental and biological risks.

## 1. Introduction

Plastic pollution has become a critical environmental issue in the 21st century, with growing recognition of its detrimental effects on ecosystems and human health [1]. While the negative impacts of larger plastic debris are well documented, recent research has focused increasingly on the dangers posed by nanoplastics—microscopic particles smaller than 100 nanometers in diameter. Due to their diminutive size, these particles are capable of penetrating biological membranes, potentially causing cellular toxicity and disrupting normal physiological functions [2]. Among the diverse range of nanoplastic particles, polystyrene nanoplastics (PS-NPs) are of particular concern [3]. This is due to the widespread use of polystyrene in various consumer products, packaging materials, and industrial applications, which increases the likelihood of environmental contamination and human exposure [4,5].

Polystyrene, which accounts for approximately 7–10% of global plastic production, is commonly used in food packaging, disposable cutlery, and consumer goods [6]. Due to its resistance to degradation, polystyrene accumulates in the environment, breaking down into polystyrene nanoplastics (PS-NPs). Studies have documented the presence of PS-NPs in marine and freshwater ecosystems [7], soil [8], and even the air [5,6]. PS-NPs can bioaccumulate in aquatic organisms and magnify through trophic levels, which may have implications for ecosystems and potential human exposure through the food chain [8].

The growing body of evidence highlights the critical need for research on polystyrene nanoplastics (PS-NPs). A 2019 study estimated that humans may ingest up to 52,000 microplastic particles annually, with nanoplastics likely contributing a significant, though not yet fully quantified, portion [9]. Animal studies further demonstrate that PS-NPs as small as 50 nanometers are capable of crossing vital biological barriers, including the blood–brain barrier, suggesting potential implications for human health [10]. Additionally, exposure to PS-NPs has been shown to adversely affect aquatic organisms, leading to reduced growth, impaired reproductive function, and changes in behavior, underscoring the broad ecological impact of these particles [11,12].

This review provides a comprehensive examination of the origins, environmental behavior, and potential health effects of PS-NPs. By synthesizing recent scientific findings, we aim to identify knowledge gaps in this rapidly developing field. This synthesis plays a pivotal role in shaping future research directions and informing policy decisions, thereby contributing to the mitigation of threats posed by these hazardous pollutants. Understanding the complexities of PS-NPs is essential for preserving environmental well-being and safeguarding human health in a world increasingly inundated with nanoplastics.

## 2. Environmental Impact and Fundamental Characteristics of PS-NPs

The journey of PS-NPs through the environment is complex. These nanoparticles spread through various pathways, such as atmospheric deposition, wastewater discharge, and the breakdown of larger plastic debris [13,14]. Their small size allows them to travel long distances, reaching even the most remote ecosystems, from deep oceans to mountain peaks [5]. In water, PS-NPs tend to clump together, affecting how they settle and how available they are to aquatic life [15]. Their long-lasting presence in the environment is concerning due to their resistance to degradation, caused by their chemical stability and the slow pace of natural breakdown. PS-NPs can also carry other pollutants, making them potentially more toxic and altering their environmental impact [16].

A clear understanding of the potential effects of PS-NPs requires insight into their characteristics and environmental behavior. The physical and chemical properties of PS-NPs determine how they interact with organisms and persist in different environments [5,8,17]. Typically, PS-NPs range in size from 1 to 100 nanometers and vary in shape and surface structure [18]. Their small size results in a higher surface area-to-volume ratio compared to larger particles, increasing their reactivity and potential for interaction with surrounding molecules.

## 3. Cellular Uptake Mechanisms of PS-NPs

PS-NPs are taken up by cells through multiple pathways, influenced by their physicochemical properties—such as size, surface modifications, and charge—as well as cell type and environmental factors. In this section, we discuss seven distinct pathways through which PS-NPs can enter cells, highlighting their complexity and diversity. The pathways include clathrin-mediated endocytosis, caveolae-mediated endocytosis, macropinocytosis, lipid raft/cholesterol-dependent endocytosis, integrin α5β1-mediated uptake, regulation by the protein corona, and the influence of lysosomes and autophagosomes.

### 3.1. Clathrin and Caveolae-Mediated Endocytosis

Clathrin-mediated endocytosis is a major pathway for PS-NP uptake, particularly in lung (A549) and colon (Caco-2) epithelial cells. Inhibiting clathrin-coated endocytosis reduces PS-NP uptake, highlighting clathrin’s role in nanoparticle internalization [19]. Additionally, caveolae-mediated endocytosis is involved in PS-NP uptake in cells with abundant caveolae, emphasizing its significance in specific cellular contexts [20].

### 3.2. Macropinocytosis

Macropinocytosis is a key pathway for PS-NP uptake, especially in cells with active cytoskeletal processes. Larger PS-NPs (>100 nm) are typically internalized via macropinocytosis [19]. Studies using macropinocytosis inhibitors, such as EIPA, have shown significant reductions in PS-NP uptake, highlighting the relevance of this pathway for larger particles [21].

### 3.3. Lipid Raft/Cholesterol-Dependent Endocytosis

Lipid raft-dependent endocytosis is a key mechanism for PS-NP uptake, especially for nanoparticles with surface modifications like amine or carboxyl groups. Cholesterol in lipid rafts is crucial for recognizing and binding nanoparticles; inhibiting cholesterol reduces PS-NP uptake, emphasizing the role of lipid rafts in cellular entry [19].

### 3.4. Integrin α5β1-Mediated Uptake

Integrin α5β1 mediates PS-NP uptake, with its overexpression significantly increasing internalization in lung epithelial cells. This enhanced uptake leads to elevated oxidative stress, mitochondrial dysfunction, and DNA damage [22]. By recognizing extracellular matrix proteins like fibronectin, integrin promotes nanoparticle uptake and amplifies intracellular oxidative stress.

### 3.5. Regulation by the Protein Corona

Under in vitro conditions, the protein corona on PS-NPs significantly affects their uptake pathways and efficiency. Factors such as plasma concentration and temperature influence the composition of the protein corona, which in turn regulates PS-NP-cell interactions. For example, a protein corona formed in high plasma concentration contains more anti-phagocytic proteins, like clustering, which reduces uptake efficiency, while a corona rich in apolipoproteins enhances cellular internalization [23].

### 3.6. Influence of Physicochemical Properties on Uptake

The size and surface charge of PS-NPs play a crucial role in determining their internalization efficiency. Smaller nanoparticles (<50 nm) are more readily internalized via clathrin-mediated pathways, while larger particles are less efficient in this regard. Positively charged PS-NPs, such as those with amine modifications, are typically internalized more efficiently due to electrostatic attraction. In contrast, negatively charged particles, like those with carboxyl modifications, tend to enter cells via lipid raft-mediated pathways [24].

### 3.7. Role of Lysosomes and Autophagosomes

PS-NPs, once internalized, frequently accumulate in lysosomes and autophagosomes, triggering autophagic responses in cells. However, these nanoparticles can also impede autophagic flux, exacerbating cellular damage [25]. The activation of autophagy and subsequent lysosomal accumulation significantly impact cellular metabolic balance and functionality, often leading to cellular stress [26].

In summary, the cellular uptake mechanisms of polystyrene nanoplastics are highly complex and diverse, governed by the physicochemical properties of the particles, the formation of the protein corona, cell type, and external environmental factors. The different uptake pathways determine the intracellular distribution of PS-NPs, which, in turn, influences their biological effects. These studies provide critical insights into the behavior of nanoplastics within biological systems, helping us better understand their potential impact on cellular health and safety.

## 4. Cellular Biological Impacts of PS-NPs

### 4.1. Oxidative Stress by PS-NPs

Exposure to PS-NPs induces oxidative stress through various mechanisms, with numerous studies reporting increased reactive oxygen species (ROS) levels across different cell types. For example, in human ovarian granulosa cells (KGN), exposure to 25 nm PS-NPs with triclosan (TCS) significantly elevated ROS levels [27]. In the mouse macrophage cell line, RAW264.7, 75–90 nm PS-NPs triggered a dose-dependent increase in ROS [28]. In human bronchial epithelial cells (BEAS-2B), exposure to 20 nm PS-NPs not only raised ROS levels but also activated calcium signaling via the ER stress pathway, worsening oxidative stress and cellular damage [29].

Chronic exposure to PS-NPs disrupts cellular redox balance by reducing antioxidant enzyme activity and increasing lipid peroxidation. In human gastric epithelial cells (GES-1), exposure to 80 nm PS-NPs significantly decreased key antioxidant enzymes (SOD, GSH-Px, CAT) and elevated malondialdehyde (MDA) levels, indicating oxidative stress [30]. Similarly, in human sperm cells, exposure to 50 nm and 100 nm PS-NPs increased ROS levels and caused significant DNA damage [31].

Furthermore, the activation of the Sirt1/ROS axis plays a pivotal role in PS-NP-induced oxidative stress. In the mouse spermatogonia cell line GC-1, PS-NP exposure led to the overexpression of the primary ROS-generating enzyme Nox2 and a concurrent downregulation of Sirt1, alongside increased levels of p53 and acetylated p53. This suggests that Sirt1 is a key regulator of oxidative stress and cellular senescence in response to PS-NP exposure [32].

Polystyrene nanoplastics (PS-NPs) induce oxidative stress through interconnected pathways, including elevated ROS production, disrupted calcium homeostasis, and impaired antioxidant enzymes. These mechanisms, observed across diverse cell types, underscore the widespread and complex impact of PS-NPs on oxidative damage.

### 4.2. Mitochondrial Damage by PS-NPs

Numerous studies have extensively investigated the mechanisms by which PS-NPs cause mitochondrial damage [33,34]. Exposure to PS-NPs in various cell types and biological models consistently leads to significant mitochondrial damage and functional impairment [35,36]. It has been shown that PS-NPs exacerbate mitochondrial damage by directly compromising mitochondrial membrane integrity and inducing oxidative stress, disrupting mitochondrial dynamics, and promoting mitophagy [37,38,39]. The specific mitochondrial damage associated with different sizes of PS-NPs includes decreased mitochondrial membrane potential (MMP), disruption of mitochondrial cristae, mitochondrial fragmentation, and significantly elevated levels of ROS [34,40]. These findings suggest that PS-NP-induced mitochondrial damage is multifaceted, affecting both mitochondrial structure and function.

More specifically, PS-NP exposure induces oxidative stress, which significantly increases mtROS levels, thereby impairing the mitochondrial antioxidant defense system and exacerbating mitochondrial membrane damage and dysfunction [35,39]. Several studies have identified excessive mtROS generation as a central mechanism of PS-NP-induced mitochondrial damage, with evidence drawn from research on intestinal epithelial cells, pulmonary epithelial cells, and renal epithelial cells [37]. Furthermore, PS-NPs significantly disturb mitochondrial dynamics by inhibiting the expression of mitochondrial fusion proteins (such as Mfn1 and Mfn2) while upregulating mitochondrial fission proteins (such as Drp1 and Fis1), leading to an imbalance in mitochondrial dynamics and excessive fragmentation [34,41]. These findings indicate that PS-NP-induced mitochondrial damage is complex, affecting both structure and function. Additionally, calcium signaling disruption further exacerbates mitochondrial stress and dysfunction [33].

In addition to oxidative stress and mitochondrial dynamics imbalance, mitophagy plays a crucial role in PS-NP-induced mitochondrial damage [38,40]. Studies involving dopaminergic neurons, renal cells, and zebrafish models have shown that exposure to PS-NPs results in the overactivation of mitophagy, primarily through the activation of the AMPK/ULK1 signaling pathway or NCOA4-mediated ferritinophagy, which further exacerbates mitochondrial damage and leads to increased cell apoptosis [35,41]. Notably, co-exposure to arsenic (As) and PS-NPs significantly aggravates mitochondrial damage and cytotoxicity by promoting mtROS-dependent ferritinophagy and ferroptosis, suggesting that PS-NPs may exhibit synergistic toxicity when combined with other environmental pollutants [33,39].

In summary, PS-NPs damage mitochondria through oxidative stress, disrupted dynamics, and overactive mitophagy. These effects are seen in many cell types and models, offering key insights into their toxic impacts. It is crucial to consider mitochondrial health when evaluating the risks of environmental nanoplastics.

### 4.3. DNA Damage by PS-NPs

Exposure to PS-NPs has been shown to cause significant DNA damage in cells. Numerous studies have demonstrated that PS-NPs induce oxidative stress, which is a major contributing factor to DNA damage [42,43]. For instance, in mouse embryonic fibroblasts, PS-NPs increase levels of ROS, leading to both direct and oxidative DNA damage. This effect is particularly severe in Ogg1-deficient cells, which cannot effectively repair oxidative damage [42]. Similarly, in human peripheral blood lymphocytes, exposure to 50 nm PS-NPs results in chromosomal aberrations and micronucleus formation, indicating that PS-NPs can cause irreversible DNA damage [14]. In GES-1, PS-NPs lead to increased ROS levels, reduced antioxidant enzyme activity, and induced both single-strand and double-strand DNA breaks, further supporting the role of oxidative stress in DNA damage [30].

The impact of PS-NPs on the cell cycle is often a downstream consequence of DNA damage. In human placental choriocarcinoma cells (JEG-3), exposure to 25–50 nm PS-NPs not only causes single-strand and double-strand DNA breaks but also results in cell cycle arrest, particularly in the G1 or G2 phases [43]. This cell cycle arrest represents a cellular response to DNA damage, activating DNA repair pathways to restore genomic stability. However, due to the excessive accumulation of ROS, these repair mechanisms are often insufficient to resolve the accumulated damage, leading to prolonged cell cycle arrest [43]. Additionally, in human neural stem cells (hNS1), increased ROS induced by PS-NPs leads to the upregulation of DNA damage-related genes such as gadd45α and rad51, while downregulating the single-strand DNA repair gene xrcc1, further exacerbating DNA damage accumulation and resulting in cell cycle arrest [44]. In mouse spermatogonia (GC-1), PS-NPs downregulate Sirt1 and increase the acetylation of p53, which exacerbates oxidative stress and DNA damage, subsequently inducing cell cycle arrest [32]. In porcine oocytes, exposure to PS-NPs increases intracellular ROS levels, with oxidative stress-induced double-strand DNA breaks being a key factor leading to cell cycle arrest between meiosis I and meiosis II [45]. In BEAS-2B, PS-NPs facilitate cellular uptake via Integrin α5β1, further exacerbating oxidative stress, which leads to DNA damage and cell cycle arrest [22].

In conclusion, PS-NPs induce oxidative stress, leading to DNA damage and subsequent cell cycle arrest through the activation of cellular DNA repair mechanisms. These mechanisms are evident across various cell types, indicating the broad and diverse impact of PS-NPs on genomic stability. When accumulated DNA damage cannot be effectively repaired, cell cycle arrest becomes an inevitable consequence, preventing damaged DNA from proceeding through further cell divisions.

### 4.4. Inflammation by PS-NPs

PS-NPs, due to their pervasive environmental presence, have emerged as a significant health concern. They have been demonstrated to induce substantial inflammatory responses in a variety of cells and organs. In HepG2 cells, exposure to PS-NPs led to a notable increase in pro-inflammatory factors such as IL-6, MCP-1, and TNF-α, displaying a clear time- and dose-dependent pattern. This elevation in inflammatory markers strongly indicates that PS-NPs can trigger significant inflammatory responses within cells [46]. Similarly, in mouse AML-12 hepatocytes, exposure to PS-NPs caused a marked increase in ROS, alongside inhibition of the NRF2 antioxidant pathway. This oxidative stress subsequently led to mitochondrial damage, which, in turn, promoted the upregulation of pro-inflammatory cytokines and activation of the NLRP3 inflammasome, ultimately inducing an inflammatory cascade [47].

Moreover, PS-NPs disrupt mitochondrial structure and dynamics, leading to both apoptosis and inflammation. In Caco-2 cells, exposure to PS-NPs in the presence of different food matrices significantly increased the expression of pro-inflammatory cytokines (such as IL-6 and IL-8) and triggered marked cellular stress and apoptosis. These effects were closely linked to mitochondrial dysfunction and elevated ROS levels [48]. Additionally, in GC-2spd spermatogenic cells, PS-NPs induced excessive mitochondrial fission and the aberrant release of mitochondrial DNA (mtDNA) into the cytoplasm, which subsequently activated the cGAS-STING signaling pathway, leading to pyroptosis. This inflammatory and pyroptotic response was particularly prominent in the group exposed to 70 nm PS-NPs [49].

Studies involving monocytes and dendritic cells showed that exposure to PS-NPs significantly increased the release of IL-6 and TNF, indicating a potent pro-inflammatory effect [50]. In mouse AML-12 hepatocytes and human L02 liver cells, exposure to various sizes of PS-NPs led to increased markers of oxidative stress and induced a pro-inflammatory response through activation of the NLRP3 inflammasome [51]. Collectively, these studies demonstrate that PS-NPs induce inflammatory responses in cells through multiple mechanisms, including oxidative stress, mitochondrial damage, activation of the cGAS-STING signaling pathway, and inflammasome activation.

In conclusion, PS-NPs trigger inflammatory responses through a variety of pathways, involving increased ROS production, mitochondrial dysfunction, and the activation of key inflammatory signaling pathways. These findings provide valuable insights into the toxic mechanisms of PS-NPs, while also highlighting the potential health risks associated with environmental exposure to these nanoplastics.

### 4.5. Autophagy Disruption by PS-NPs

PS-NPs exert a profound influence on autophagy regulation through multiple mechanisms, with lysosomal dysfunction playing a particularly crucial role. PS-NPs not only activate autophagic responses but also interfere with autophagic flux via diverse signaling pathways. In mouse embryonic fibroblasts (MEFs), PS-NPs penetrate the cytoplasm and lead to the accumulation of autophagosomes. The inability of lysosomes to effectively degrade these autophagosomes results in their continued accumulation, culminating in lysosomal dysfunction [52]. Similarly, in primary human nasal epithelial cells (HNEpCs), exposure to 50 nm PS-NPs induces oxidative stress and mitochondrial dysfunction, thereby hindering autophagic flux and promoting the accumulation of autophagosomes [53].

Moreover, in human liver cells (L-02 cells), PS-NPs trigger lipophagy by activating the AMPK/ULK1 signaling pathway. However, lysosomal dysfunction impairs autophagic flux, leading to lipid droplet accumulation within hepatocytes [54]. This phenomenon was also observed in HepG2, where exposure to 100 nm PS-NPs stimulated autophagosome formation through the ERK/mTOR signaling pathway. Despite this activation, weakened lysosomal transport and hydrolase functions led to ineffective autophagosome degradation [55]. In a similar manner, oxidative stress induced by PS-NPs in mouse spermatogonia-derived GC-2spd(ts) cells compromised lysosomal membrane integrity, thereby disrupting normal autophagic flux [56].

Neutral Red assay results further corroborate the significant impact of PS-NPs on lysosomal function. In studies using 3T3 fibroblast cells, exposure to PS-NPs revealed no significant lysosomal dysfunction under low concentrations. However, at higher concentrations or under oxidative stress conditions, lysosomal activity was noticeably impaired, as indicated by reduced Neutral Red uptake [57]. Furthermore, studies with A549 cells showed that PS-NPs localized primarily within lysosomes and endoplasmic reticulum compartments. Despite this localization, Neutral Red assay results demonstrated that lysosomal damage was evident, particularly under prolonged or high-dose exposures [58]. These findings highlight the dual role of PS-NPs in modulating lysosomal integrity and autophagic flux.

Several studies have also highlighted the downregulation of lysosomal membrane proteins, such as LAMP1 and LAMP2, as well as hydrolases like CTSB and CTSD. These alterations further hinder the fusion and degradation processes of autophagosomes and lysosomes [25,53]. In mouse granulosa cells, 25 nm PS-NPs activate autophagy by inhibiting the PI3K/AKT signaling pathway, yet lysosomal dysfunction impairs autophagic flux, preventing autophagosome degradation and ultimately leading to cell apoptosis [59].

In conclusion, PS-NPs significantly affect autophagy regulation across various cell types by inducing autophagy activation while simultaneously causing lysosomal damage. Lysosomal dysfunction emerges as a central mechanism by which PS-NPs interfere with autophagic flux, impacting lysosomal membrane integrity, lysosomal hydrolases, and the fusion process between autophagosomes and lysosomes. These findings underscore the complexity of PS-NPs’ effects on autophagy regulation, particularly emphasizing the critical role of lysosomal damage in obstructing autophagic flux, which may be a key factor underlying their biological toxicity.

### 4.6. Other Biological Effects of PS-NPs

After exploring the effects of PS-NPs on oxidative stress, mitochondrial damage, DNA damage, inflammation, and autophagy, it is crucial to also consider other significant cellular consequences, such as endoplasmic reticulum (ER) stress and cell membrane damage. ER stress is a pivotal mechanism contributing to cellular toxicity, often triggered when PS-NPs activate ROS-dependent pathways. This activation subsequently initiates the unfolded protein response (UPR) and upregulates the expression of key ER stress-related genes, including GRP78, ATF6, PERK, and CHOP [60]. Moreover, studies involving the mouse macrophage cell line RAW264.7 have demonstrated that PS-NPs with various surface functionalities can induce ER stress, with amino-functionalized PS-NH2 eliciting a particularly pronounced response, as indicated by elevated expression of ER stress markers [61]. These findings suggest that PS-NPs can induce ER stress through multiple mechanisms, ultimately compromising cellular homeostasis and function.

In addition to ER stress, cell membrane damage represents another major consequence of PS-NP exposure. Lactate dehydrogenase (LDH) is a well-established and widely utilized biomarker for evaluating cell membrane damage. Studies have shown that exposure to 100 nm and 500 nm PS-NPs at a concentration of 25 μg/mL for 48 h significantly elevated LDH activity in the supernatants of human umbilical vein endothelial cell (HUVEC) cultures, indicating substantial disruption to cell membrane integrity [62]. Moreover, smaller amino-modified PS-NPs (20 nm) exhibited notably higher toxicity in plant protoplasts, as evidenced by a pronounced increase in extracellular LDH levels. In contrast, larger particles had a comparatively minimal effect. These findings highlight the heightened disruptive potential of smaller PS-NPs on cell membranes [63]. Furthermore, PS-NP exposure has been shown to impair cellular lipid metabolism, particularly in human macrophages, where it promotes the accumulation of lipid droplets within the cytoplasm and fosters macrophage differentiation into foam cells [64].

In summary, PS-NPs have notable detrimental effects on cellular biology, including increased oxidative stress, mitochondrial dysfunction, DNA damage, inflammation, and disrupted autophagy. PS-NPs exert their toxicity by overproducing reactive ROS, leading to mitochondrial imbalance, abnormal autophagy, and the activation of inflammation-related pathways. Additionally, PS-NPs induce endoplasmic reticulum stress and compromise cell membrane integrity, which collectively impairs cellular function. The various biological effects of PS-NPs on different cell types are summarized in Table 1, providing a concise overview of their widespread impact on cellular health and function.

## 5. Cell Death Induced by **PS-NPs**

**Figure 1:**
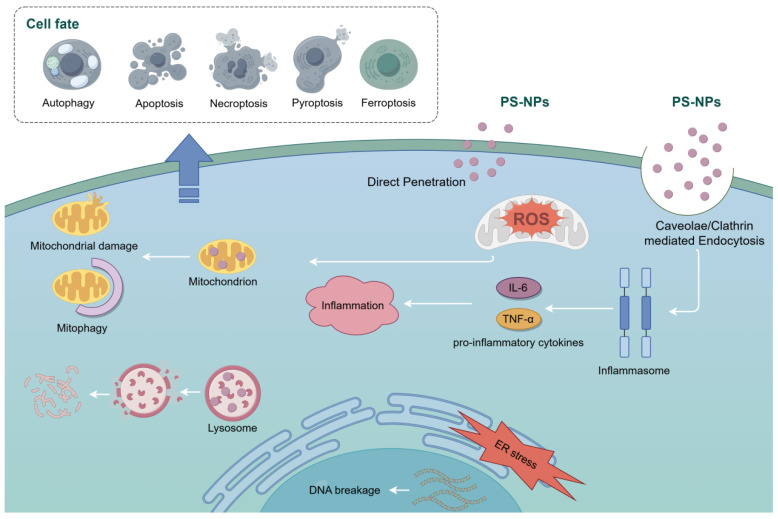
Mechanistic overview of PS-NP-induced cellular impacts. This figure illustrates the key biological impacts of PS-NPs on cellular systems, including oxidative stress, mitochondrial dysfunction, DNA damage, inflammation, autophagy disruption, and various forms of cell death.

The cellular biological impacts of PS-NPs are intricate and involve a cascade of detrimental effects on cellular systems. Key impacts include oxidative stress, mitochondrial dysfunction, DNA damage, inflammation, and disruptions in autophagy, all of which contribute to various forms of cell death, such as apoptosis, ferroptosis, necroptosis, and pyroptosis (Figure 1).

### 5.1. Apoptosis Induced by PS-NPs

PS-NPs induce apoptosis through multiple pathways, causing significant tissue and organ damage. In various cell types, PS-NPs provoke oxidative stress, mitochondrial dysfunction, inflammatory responses, and upregulation of pro-apoptotic proteins, leading to cell death. In Caco-2 cells, co-exposure to PS-NPs and okadaic acid (OA) intensified ER stress, triggering apoptosis via the PERK/ATF-4/CHOP pathway and causing mitochondrial membrane depolarization with calcium overload [65]. Similarly, in HepG2 human liver cancer cells, PS-NPs caused mitochondrial damage, reduced ATP synthesis, diminished mitochondrial membrane potential, and increased ROS levels, promoting Caspase-3 upregulation [66]. In murine Sertoli cells, PS-NP exposure lowered anti-apoptotic Bcl-2 levels, upregulated pro-apoptotic Bax, and activated the TLR4 signaling pathway, amplifying inflammation and apoptosis [67].

Furthermore, PS-NPs induced apoptosis in Sertoli cells by disrupting the integrity of the blood–testis barrier (BTB). In a murine model, exposure to 80 nm PS-NPs compromised BTB integrity, causing a notable decline in the expression of barrier-associated proteins such as occludin and ZO-1, which subsequently precipitated apoptosis in Sertoli cells [67]. In KGN human ovarian granulosa cells, PS-NP exposure significantly heightened oxidative stress, resulting in the activation of the NRF2-Keap1-HO-1 signaling pathway and leading to the increased expression of the pro-apoptotic protein Caspase-3 [27]. In RAW264.7 murine macrophages, PS-NPs activated the cGAS-STING signaling pathway, thereby promoting the release of inflammatory cytokines and enhancing the expression of pro-apoptotic proteins [68]. Collectively, these findings illustrate that PS-NPs induce apoptosis through diverse mechanisms—including oxidative stress, inflammatory cascades, signaling pathway activation, and barrier disruption—underscoring their toxicological impact across different cellular contexts and their broader implications for biological systems.

### 5.2. Ferroptosis Induced by PS-NPs

Ferroptosis has been shown to play critical roles in environmental stimulus-mediated toxicity [69,70]. PS-NPs have been shown to induce ferroptosis through a variety of mechanisms, which have been thoroughly studied across multiple cell types. For instance, in human bronchial epithelial cells, exposure to PS-NPs leads to a reduction in glutathione (GSH) levels and an increase in reactive ROS, accompanied by significant downregulation of ferritin (FTH1) and glutathione peroxidase 4 (GPX4), ultimately resulting in ferroptosis [71]. Similarly, in the human pulmonary artery and bronchial epithelial cells, PS-NPs trigger ferritinophagy, which releases free iron, leading to lipid peroxidation and subsequent ferroptosis [36].

While the specific pathways of PS-NP-induced ferroptosis can vary slightly between cell types, they are consistently linked to oxidative stress and disrupted iron metabolism. For example, in HepG2, PS-NP exposure causes iron overload and impaired antioxidant enzyme activity, characterized by reduced levels of GPX4 and ferritin along with increased lipid peroxidation, indicative of ferroptosis [46]. In mouse brain microvascular endothelial cells (bEnd.3), PS-NPs upregulate the transferrin receptor (TFRC) while downregulating GPX4, which exacerbates blood–brain barrier damage [72].

Additionally, PS-NPs can induce ferroptosis via the endoplasmic ER stress pathway. In human bronchial epithelial cells, PS-NP exposure activates the IRE1α and PERK signaling pathways, resulting in increased ROS production and lipid peroxidation, both of which are linked to ferroptosis [29]. In GC-2, PS-NPs induce ferroptosis by affecting the Nrf2 signaling pathway and related iron metabolism proteins, including transferrin receptor (TFR) and divalent metal transporter 1 (DMT1) [73].

In grass carp hepatocytes and porcine oocytes, PS-NPs induce ferroptosis through mechanisms involving iron metabolism dysregulation, iron overload, and lipid peroxidation, along with changes in the expression of ferritinophagy-related proteins such as NCOA4 and antioxidant enzymes like GPX4 [45,74]. Notably, in primary hippocampal neurons from neonatal rats, PS-NP exposure induces ferroptosis via P53-mediated ferritinophagy, further contributing to neuronal damage [75].

In summary, PS-NPs induce ferroptosis through multiple interconnected mechanisms, including oxidative stress, disruption of iron metabolism, ER stress activation, and ferritinophagy. These mechanisms have been observed across a range of cell types, highlighting the broad and diverse toxic effects of PS-NPs. These findings underscore the need to consider ferroptosis as a critical factor in future environmental and health risk assessments of nanoplastic exposure.

### 5.3. Necroptosis and Pyroptosis Induced by PS-NPs

In addition to apoptosis and ferroptosis, PS-NPs also induce other forms of cell death, notably necroptosis, and pyroptosis, each characterized by distinct yet interrelated mechanisms. Necroptosis is prominently triggered by PS-NPs through the activation of receptor-interacting protein kinases (RIPK1 and RIPK3), often associated with mitochondrial dysfunction and increased ROS. For instance, in human brain microvascular endothelial cells (hCMEC/D3), PS-NP exposure activated the RIPK3/MLKL signaling pathway, leading to necroptosis via elevated ROS production and NF-κB activation [10]. Similarly, in mouse intestinal epithelial cells, PS-NPs caused necroptosis by impairing lysosomal function and disrupting mitochondrial dynamics, resulting in the activation of the PINK1/Parkin-mediated mitophagy pathway, but subsequently blocking its completion, which ultimately led to RIPK3/MLKL-mediated cell death [40]. In HepG2 cells, PS-NP exposure further demonstrated necroptotic effects, as evidenced by an increase in phosphorylated RIPK3 [76].

Pyroptosis, an inflammatory form of programmed cell death [77], is also a significant outcome of PS-NP exposure. This pathway typically involves inflammasome activation and the subsequent cleavage of gastrin D (GSDMD), which results in cell membrane rupture and the release of pro-inflammatory contents. In murine lung epithelial cells (MLE-12), amine-functionalized PS-NPs (APS-NPs) induced pyroptosis by activating the NLRP3 inflammasome and Caspase-1, leading to the release of inflammatory cytokines, such as IL-1β and TNF-α [78]. In mouse liver tissues, PS-NPs were shown to activate the TLR4/NF-κB/NLRP3/GSDMD pathway, resulting in increased levels of inflammatory cytokines and pyroptosis-associated proteins, suggesting a connection between intestinal barrier disruption and liver inflammation [79]. In another study involving mouse cardiomyocytes, co-exposure to PS-NPs and cadmium (Cd) led to both pyroptosis and necroptosis, a phenomenon referred to as PANoptosis, highlighting a complex interplay of multiple cell death pathways in response to nanoparticle exposure [80]. Similarly, in HepG2 cells, PS-NP exposure activated pyroptosis through NLRP3 inflammasome signaling, characterized by increased levels of Caspase-1, GSDMD, IL-1β, and IL-18 [76].

These findings collectively demonstrate that PS-NPs not only induce classical apoptosis but also initiate diverse and often overlapping cell death pathways, including necroptosis and pyroptosis. Such multifaceted effects underscore the broad and potentially harmful impact of PS-NPs on various tissues and organs.

**Figure 2:**
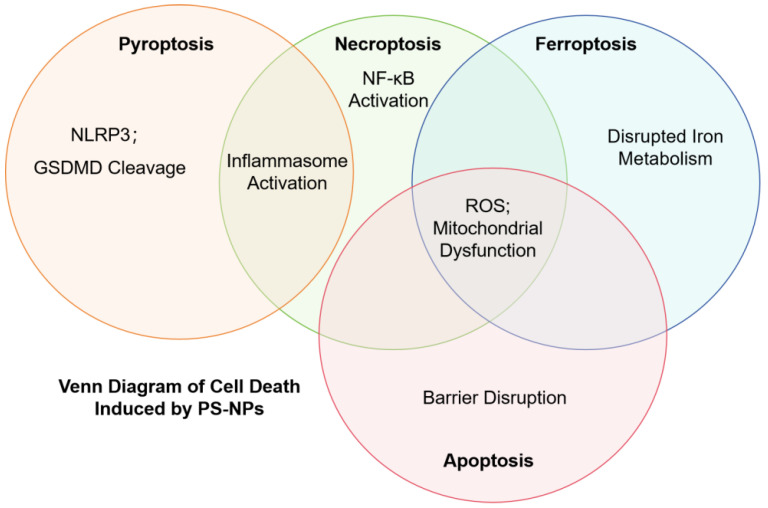
Venn diagram of key cell death pathways induced by PS-NPs. Venn diagram illustrating the key pathways involved in different forms of cell death induced by PS-NPs. The diagram shows the overlapping mechanisms of apoptosis, ferroptosis, necroptosis, and pyroptosis.

In summary, PS-NPs cause significant cellular damage through multiple mechanisms, including oxidative stress, mitochondrial dysfunction, DNA damage, and inflammation. They disrupt autophagy, activate inflammatory pathways, and induce endoplasmic reticulum stress, leading to compromised cell membrane integrity and impaired cellular functions. These biological effects are summarized in Table 2 and illustrated in Figure 2, offering a comprehensive overview of the key signaling pathways and types of cell death induced by PS-NPs across various cell types.

## 6. Factors Influencing the Cytotoxicity of PS-NPs

The cytotoxicity of PS-NPs is influenced by various interrelated factors, including co-exposure to other pollutants, particle size, surface functional groups, electric charge, shape, aging, protein corona formation, exposure time, and exposure conditions. These factors interact in complex ways to shape the overall toxicity profile.

### 6.1. Co-Exposure to Pollutants

Co-exposure with other pollutants often markedly intensifies the toxic effects of PS-NPs. Numerous studies have demonstrated that PS-NPs, when co-exposed with heavy metals (such as arsenic and cadmium), antibiotics (such as norfloxacin), or environmental contaminants (like silver nanoparticles), exhibit significant synergistic toxicity. This co-exposure exacerbates oxidative stress, inflammatory responses, and DNA damage, leading to increased cytotoxicity [34,81,82]. For instance, co-exposure of PS-NPs with arsenic significantly induces ferroptosis, evident through mitochondrial dysfunction and heightened oxidative stress [34].

### 6.2. Particle Size

Particle size is another critical determinant of PS-NP toxicity. Research has shown that smaller PS-NPs (e.g., 20 nm to 100 nm) are more easily internalized by cells compared to their larger counterparts. Their larger specific surface area facilitates stronger interactions with cellular structures, resulting in greater toxicity [81,83]. Smaller particles have a pronounced ability to induce oxidative stress, mitochondrial damage, and DNA damage, particularly in murine and human cell lines [83].

### 6.3. Surface Functional Groups

Surface functional groups significantly influence the toxic effects of PS-NPs. Amino-functionalized PS-NPs (PS-NH2) exhibit a heightened toxicity compared to carboxyl-functionalized (PS-COOH) or non-functionalized PS-NPs, primarily due to the strong electrostatic interactions between the positively charged PS-NH2 and the negatively charged cell membrane. This results in increased cellular uptake and a heightened cytotoxic effect.

### 6.4. Electric Charge

The electric charge of PS-NPs also plays a pivotal role in determining their biological effects. Positively charged PS-NPs exhibit stronger interactions with the negatively charged phospholipid components of cell membranes, which promotes higher internalization rates and increased cytotoxicity. For example, positively charged PS-NH2 particles induce significantly more oxidative stress and apoptosis compared to negatively charged, carboxyl-functionalized PS-NPs [61].

### 6.5. Shape

The shape of PS-NPs is another key factor influencing their cytotoxic effects. Spherical PS-NPs tend to be more readily internalized via endocytosis, leading to greater intracellular accumulation and disruption of cellular functions [84]. In contrast, irregularly shaped PS-NPs may adhere to cell surfaces for longer periods, altering their interaction dynamics and, consequently, their toxicity [84].

### 6.6. Aging

Aging further amplifies the toxicity of PS-NPs. Ultraviolet (UV) aging changes the surface properties of PS-NPs, such as increasing the number of oxidized functional groups, which heightens hydrophilicity and strengthens their interactions with biological systems. Studies have shown that aged PS-NPs, particularly when co-exposed with heavy metals like lead, exhibit significantly increased toxicity due to enhanced metal adsorption capabilities brought on by surface modifications [82].

### 6.7. Protein Corona Formation

Finally, protein corona formation substantially modulates PS-NP toxicity. When PS-NPs are exposed to biological fluids, they swiftly adsorb proteins to form a “protein corona”. This layer of proteins effectively masks the native surface characteristics of PS-NPs, reducing their direct interaction with cell membranes. Consequently, the presence of a protein corona generally lowers the toxicity of PS-NPs by reducing cellular uptake and subsequent intracellular damage [85].

### 6.8. Exposure Time

While most studies have focused on short-term acute exposures, long-term low concentration may more accurately reflect real-world environmental conditions. For instance, eight weeks of continuous exposure in Caco-2 cells demonstrated minimal acute cytotoxicity but induced mild chronic stress, including upregulation of stress-related genes such as HO1 and SOD2. PS-NPs were found to accumulate inside cells in a concentration-dependent manner, stabilizing after two weeks [86]. These observations suggest that prolonged exposure, though not acutely toxic, can gradually alter cellular behavior. This highlights the critical need to investigate chronic exposure scenarios further. However, research in this area remains limited, and future studies should explore diverse cell models, extended exposure periods, and interactions with environmental stressors to better elucidate the long-term impacts of PS-NPs.

### 6.9. Exposure Conditions

The exposure method plays a pivotal role in determining the cytotoxic effects of PS-NPs. Advances in vitro models, such as co-culture systems and air–liquid interface (ALI) cultures, offer physiologically relevant conditions that better mimic real-world scenarios compared to traditional submerged cultures.

Co-culture models provide a sophisticated framework for studying the interactions between nanoparticles and complex multicellular environments. For instance, a co-culture system comprising Caco-2 and Raji cells effectively simulates the intestinal follicle-associated epithelium, facilitating investigations into nanoparticle transport mechanisms like macropinocytosis in M cells [87]. Similarly, co-cultures of Caco-2 and HT29-MTX cells replicate the intestinal barrier’s functionality, demonstrating that while high doses of PS-NPs result in minimal translocation across the epithelial layer (<10%), the barrier’s integrity remains intact [88]. Furthermore, exposure conditions such as gastrointestinal enzymatic pre-digestion significantly enhance the translocation of PS-NPs through intestinal co-culture models, highlighting the importance of exposure scenarios in evaluating nanoparticle toxicity [89].

ALI cultures provide a realistic representation of respiratory exposure to PS-NPs, capturing key aspects of aerosolized particle interactions with airway cells. Studies comparing ALI and submerged culture systems reveal that aerosolized PS-NPs under ALI conditions induce significantly higher cytotoxicity, attributed to the direct and localized interaction of nanoparticles with the cellular surface [90]. Using advanced systems like the Vitrocell^®^ Cloud, research has demonstrated distinct penetration behaviors of aerosolized PS-NPs under ALI conditions, emphasizing the necessity of replicating realistic exposure scenarios to accurately assess nanoparticle toxicity in respiratory models [91].

Collectively, these advanced models underscore the critical importance of physiologically relevant exposure conditions when assessing the cytotoxicity of PS-NPs. Co-culture and ALI systems not only provide nuanced insights into nanoparticle transport, barrier interactions, and cytotoxic outcomes but also serve as indispensable tools for bridging the gap between experimental conditions and real-world exposures.

## 7. Challenges in Detecting PS-NPs

The detection and analysis of PS-NPs pose significant challenges due to their small size, low concentrations in the environment, and the complexity of sample matrices. Despite recent advancements, the lack of standardized methodologies continues to hinder progress in understanding their environmental and biological impacts.

### 7.1. Sensitivity and Resolution

Commonly used techniques, such as Dynamic Light Scattering (DLS) and Nanoparticle Tracking Analysis (NTA), are effective for particle size determination. However, these methods are insufficient for distinguishing plastic particles from natural ones without additional chemical characterization [92]. Advanced tools, including Raman microscopy and Fourier-Transform Infrared (FTIR) spectroscopy, provide greater sensitivity but are susceptible to fluorescence interference from sample matrices, limiting their utility in complex environmental samples [93].

### 7.2. Matrix Interference

Nanoplastics often aggregate in response to ionic strength variations in solutions, complicating their separation and detection. Furthermore, the presence of pigments or organic matter in environmental samples generates fluorescence noise that interferes with spectroscopic analyses. These issues necessitate the development of advanced noise suppression techniques and novel methods to prevent aggregation [93].

### 7.3. Separation and Characterization

Techniques such as ultrafiltration and continuous-flow centrifugation are widely used for nanoplastic separation but are time-intensive and require careful optimization, particularly for samples with low concentrations. Methods like Pyrolysis–Gas Chromatography–Mass Spectrometry (Py-GC-MS) effectively identify polymers but are destructive and require high-purity samples, limiting their broader application [92,94].

### 7.4. Lack of Standardization

The absence of harmonized protocols for sampling, pretreatment, and analysis is one of the most pressing challenges. Variability in filtration techniques, calibration standards, and pretreatment methods leads to inconsistencies across studies, reducing the reproducibility of findings. Establishing standardized procedures and reference materials is critical for enabling reliable cross-study comparisons and advancing the field [94].

### 7.5. Emerging Techniques

Emerging approaches, such as Surface-Enhanced Raman Spectroscopy (SERS) and integrated multi-instrumental methods combining Raman microscopy with X-ray Photoelectron Spectroscopy (XPS) or Scanning Electron Microscopy (SEM), show promise in addressing current analytical limitations. However, these methods remain in the early stages of development and require extensive validation to demonstrate their applicability in real-world scenarios [93].

### 7.6. Recommendations for Standardization and Improvement

Developing Harmonized Protocols: Establish unified guidelines for nanoplastic sampling, identification, and quantification to ensure consistency and reproducibility across studies [94].

Advancing Interference Mitigation: Focus on enhancing techniques to suppress matrix fluorescence and other spectroscopic interferences [93].

Standardizing Reference Materials: Create universally accepted reference standards to facilitate cross-study comparisons and method validation [94].

Promoting Collaborative Efforts: Encourage collaboration among researchers, regulatory bodies, and standardization organizations (e.g., ISO, ASTM) to drive the establishment of globally accepted methodologies.

## 8. Conclusions

In summary, the biological effects of PS-NPs on cellular systems are diverse and profound, encompassing oxidative stress, mitochondrial dysfunction, DNA damage, inflammation, and disruptions in cellular processes like autophagy and apoptosis. PS-NPs induce multiple forms of cell death, including apoptosis, ferroptosis, necroptosis, and pyroptosis, each mediated through distinct yet interconnected molecular mechanisms. Furthermore, factors such as particle size, surface modifications, exposure duration and conditions, co-exposure with other pollutants, and the presence of protein coronas significantly modulate the cytotoxicity of PS-NPs, adding layers of complexity to their biological impact. These findings underscore the urgent need for continued research to unravel the exact pathways and health implications associated with PS-NP exposure, particularly in light of their pervasive presence in the environment and their potential to accumulate in biological systems.

Beyond these biological effects, the detection and analysis of PS-NPs present significant technical challenges due to their nanoscale dimensions, low environmental concentrations, and the complexity of the matrices in which they are found. Current methodologies, such as spectroscopic and chromatographic techniques, are often limited by issues like fluorescence interference, particle aggregation, and a lack of standardized protocols. Emerging technologies, including Surface-Enhanced Raman Spectroscopy (SERS) and multi-instrumental approaches, hold promise in overcoming these limitations but require extensive validation before they can be widely applied in real-world scenarios. Addressing these analytical hurdles is essential for advancing our understanding of the environmental and biological impacts of PS-NPs and guiding future research efforts.

Furthermore, it is important to acknowledge that most existing studies on PS-NP toxicity rely on commercial PS latexes, which may not accurately reflect the properties of nanoplastics produced in the environment. The physical and chemical differences between these commercial materials and environmentally relevant PS-NPs could lead to variations in toxicity profiles, emphasizing the need for future studies to focus on materials that better represent real-world conditions. Moreover, the widespread use of extremely high PS-NP concentrations in experimental studies, while valuable for exploring mechanisms, may not fully capture the risks posed by typical environmental exposure levels. This disconnect underscores the importance of designing studies that incorporate realistic exposure scenarios to improve the relevance of findings for public health assessments.

Taken together, these findings highlight the urgent need for continued research to unravel the pathways and health implications of PS-NP exposure. Future studies should prioritize understanding the long-term effects of PS-NPs on human health, investigating their molecular interactions, and developing strategies to mitigate their impact. Equally crucial is the advancement of standardized protocols for sampling, detection, and analysis, alongside the development of interference mitigation techniques and universally accepted reference standards. Collaborative efforts among researchers, regulatory bodies, and standardization organizations will be pivotal in overcoming these challenges, ensuring reliable cross-study comparisons, and ultimately reducing the risks posed by environmental nanoplastics.

## Figures and Tables

**Table 1:** Representative studies on the biological effects of PS-NPs on cells.

Biological Effect	Cell Type	PS-NPs Size (nm)	Effect Observed	Source
Oxidative Stress	KGN	25	Increased ROS, decreased SOD and CAT activity, elevated MDA, mitochondrial dysfunction, activation of NRF2-KEAP1-HO-1 pathway, inhibition of SESTRIN2.	[25]
	RAW264.7	70, 90	Induced oxidative stress, inflammation.	[26]
	BEAS-2B	20	Increased ROS, elevated MDA, ROS-dependent ER stress.	[27]
	GES-1	80	Reduced antioxidant enzyme activity, increased MDA, 8-OhdG, γ-H2AX, redox-dependent activation of β-catenin/YAP.	[28]
Mitochondrial Damage	VSMCs	100	Overexpression of ROS, accumulation of mutated mtDNA, dysregulation of genes related to mitochondrial synthesis and division.	[31]
	HPAEpiC BEAS-2B	50	Disturbance of mitochondrial structure and function, oxidative stress-derived mitochondrial damage.	[34]
	Caco-2	100	Mitochondrial stress, activation of PINK1/Parkin-mediated mitophagy, blockade of mitophagic flux, and induction of necroptosis.	[38]
DNA Damage	BEAS-2B	80	Induction of oxidative stress due to mitochondrial dysfunction, leading to DNA damage and inflammation.	[21]
	GC-1	80	ROS-induced oxidative stress, activation of senescence-related signaling pathways (p53-p21/Rb-p16), and subsequent DNA damage.	[30]
	MEF	50	Physical interaction between pollutants enhances oxidative DNA damage, leading to cell phenotype transformation.	[40]
	JEG-3	25, 50	Increased ROS, DNA damage, cell cycle arrest (G1/G2).	[41]
	HhNS1	30	Induced oxidative stress and cellular stress, leading to DNA damage, alterations in the inflammatory response, and apoptosis.	[42]
Inflammation	HepG2	40	Increased ROS, activation of NF-κB pathway, and release of pro-inflammatory cytokines (IL-6, TNF-α).	[44]
	GC-2spd	70	Generation of ROS, inhibition of NRF2, activation of NLRP3 inflammasome, increased IL-1β, caspase-1, and NF-κB activation.	[45]
	RAW264.7 AML-12	75, 90 20	Activated cGAS-STING pathway, upregulation of inflammation-related factors (NLRP3, ASC, Caspase1 p20, IL-1β).	[47]
Autophagy	HIEC-6	100	Impaired autophagic flux, triggering of autophagy.	[24]
	hESCs	20, 100	Increased iROS, decreased MMP, accumulation of LC3-II and p62, indicating defective autophagy.	[51]
	HepG2	100	PS-NPs activate autophagosome formation via ERK/mTOR, but impaired lysosomal function blocks autophagic flux, contributing to lipid accumulation.	[53]
Endoplasmic Reticulum Stress	BEAS-2B	20	Induced ER stress, protein misfolding, activation of UPR, upregulation of ubiquitin ligases (HRD1, CHIP).	[56]
	RAW264.7	100	Induced ER stress, protein misfolding, oxidative stress, calcium imbalance, activation of UPR.	[57]
Membrane Damage	HUVECs	100	Caused cell membrane damage through internalization and aggregation, leading to lactate dehydrogenase release.	[58]

**Table 2:** Summary of key signaling pathways in PS-NP-induced cell death.

Cell Death Type	Signaling Pathway	Signaling Molecule	Observed Cell Type	Source
Apoptosis	PERK/ATF-4 /CHOP	ROS, Calcium overload, Bax/Bcl-2 ratio, Caspase-3	Caco-2	[63]
	TLR4	ROS, Bax/Bcl-2, Caspase-3	HepG2	[64]
	Caspase-3	ROS, Caspase-3	Sertoli cells	[65]
	NRF2-Keap1-HO-1	ROS, Caspase-3	KGN cells	[25]
	cGAS-STING	Inflammatory cytokines, Caspase-3	RAW264.7	[66]
Ferroptosis	IRE1α	ROS, GPX4, Ferritin (FTH1), Iron overload	BEAS-2b	[67]
	PERK	ROS, GPX4, Ferritin (FTH1)	HepG2	[44]
	Nrf2	GPX4, TFRC, DMT1	GC-2 cells	[69]
	Transferrin receptor (TFRC)	ROS, GPX4, Ferritin (FTH1)	bEnd.3	[68]
		Lipid peroxidation, Ferritinophagy	Porcine oocytes	[70]
		ROS, Lipid peroxidation, Ferritinophagy	Hippocampal neurons,	[71]
Necroptosis	RIPK1, RIPK3, MLKL	ROS, NF-κB, Phosphorylated RIPK3	hCMEC/D3	[9]
	PINK1/Parkin	ROS, Mitophagy	IEC	[38]
		ROS, Phosphorylated RIPK3	HepG2	[72]
Pyroptosis	NLRP3 inflammasome	Caspase-1, IL-1β, TNF-α, IL-18, GSDMD	MLE-12 cells	[73]
		Caspase-1, GSDMD, IL-18	Mouse cardiomyocytes	[75]
		IL-1β, TNF-α, GSDMD	HepG2	[72]

## Data Availability

No new data were created or analysed in this study. Data sharing is not applicable to this article.

## References

[1] Kumar M., Chen H., Sarsaiya S., Qin S., Liu H., Awasthi M.K., Kumar S., Singh L., Zhang Z., Bolan N.S. (2021). Current research trends on micro- and nano-plastics as an emerging threat to global environment: A review. J. Hazard. Mater..

[2] Jiang W., Hu C., Chen Y., Li Y., Sun X., Wu H., Yang R., Tang Y., Niu F., Wei W. (2023). Dysregulation of the microbiota-brain axis during long-term exposure to polystyrene nanoplastics in rats and the protective role of dihydrocaffeic acid. Sci. Total Environ..

[3] Sui A., Yao C., Chen Y., Li Y., Yu S., Qu J., Wei H., Tang J., Chen G. (2023). Polystyrene nanoplastics inhibit StAR expression by activating HIF-1α via ERK1/2 MAPK and AKT pathways in TM3 Leydig cells and testicular tissues of mice. Food Chem. Toxicol..

[4] Di M., Wang J. (2018). Microplastics in surface waters and sediments of the Three Gorges Reservoir, China. Sci. Total Environ..

[5] Allen S., Allen D., Phoenix V.R., Le Roux G., Jiménez P.D., Simonneau A., Binet S., Galop D. (2019). Atmospheric transport and deposition of microplastics in a remote mountain catchment. Nat. Geosci..

[6] Amobonye A., Bhagwat P., Raveendran S., Singh S., Pillai S. (2021). Environmental Impacts of Microplastics and Nanoplastics: A Current Overview. Front. Microbiol..

[7] Mattsson K., Hansson L.A., Cedervall T. (2015). Nano-plastics in the aquatic environment. Environ. Sci. Process Impacts.

[8] Kik K., Bukowska B., Sicińska P. (2020). Polystyrene nanoparticles: Sources, occurrence in the environment, distribution in tissues, accumulation and toxicity to various organisms. Environ. Pollut..

[9] Cox K.D., Covernton G.A., Davies H.L., Dower J.F., Juanes F., Dudas S.E. (2019). Human Consumption of Microplastics. Environ. Sci. Technol..

[10] Shan S., Zhang Y., Zhao H., Zeng T., Zhao X. (2022). Polystyrene nanoplastics penetrate across the blood-brain barrier and induce activation of microglia in the brain of mice. Chemosphere.

[11] Bergami E., Pugnalini S., Vannuccini M.L., Manfra L., Faleri C., Savorelli F., Dawson K.A., Corsi I. (2017). Long-term toxicity of surface-charged polystyrene nanoplastics to marine planktonic species Dunaliella tertiolecta and Artemia franciscana. Aquat. Toxicol..

[12] Zhu C., Liu G., Abdullah A.L.B., Han M., Jiang Q., Li Y. (2023). Transcriptomic analysis following polystyrene nanoplastic stress in the Pacific white shrimp, Litopenaeus vannamei. Fish. Shellfish. Immunol..

[13] Lambert S., Wagner M. (2016). Characterisation of nanoplastics during the degradation of polystyrene. Chemosphere.

[14] Sarma D.K., Dubey R., Samarth R.M., Shubham S., Chowdhury P., Kumawat M., Verma V., Tiwari R.R., Kumar M. (2022). The Biological Effects of Polystyrene Nanoplastics on Human Peripheral Blood Lymphocytes. Nanomaterials.

[15] Guo Y., Tang N., Lu L., Li N., Hu T., Guo J., Zhang J., Zeng Z., Liang J. (2024). Aggregation behavior of polystyrene nanoplastics: Role of surface functional groups and protein and electrolyte variation. Chemosphere.

[16] Guo S., Shi H., Qi Y., Tian G., Wang T., He F., Li X., Liu R. (2023). Environmental relevant concentrations of polystyrene nanoplastics and lead co-exposure triggered cellular cytotoxicity responses and underlying mechanisms in Eisenia fetida. Sci. Total Environ..

[17] Teng M., Zhao X., Wu F., Wang C., Wang C., White J.C., Zhao W., Zhou L., Yan S., Tian S. (2022). Charge-specific adverse effects of polystyrene nanoplastics on zebrafish (Danio rerio) development and behavior. Environ. Int..

[18] Paul M.B., Stock V., Cara-Carmona J., Lisicki E., Shopova S., Fessard V., Braeuning A., Sieg H., Böhmert L. (2020). Micro- and nanoplastics—Current state of knowledge with the focus on oral uptake and toxicity. Nanoscale Adv..

[19] Liu Y.Y., Liu J., Wu H., Zhang Q., Tang X.R., Li D., Li C.S., Liu Y., Cao A., Wang H. (2022). Endocytosis, Distribution, and Exocytosis of Polystyrene Nanoparticles in Human Lung Cells. Nanomaterials.

[20] Monti D.M., Guarnieri D., Napolitano G., Piccoli R., Netti P., Fusco S., Arciello A. (2015). Biocompatibility, uptake and endocytosis pathways of polystyrene nanoparticles in primary human renal epithelial cells. J. Biotechnol..

[21] Phuc L.T.M., Taniguchi A. (2017). Epidermal Growth Factor Enhances Cellular Uptake of Polystyrene Nanoparticles by Clathrin-Mediated Endocytosis. Int. J. Mol. Sci..

[22] Han M., Liang J., Wang K., Si Q., Zhu C., Zhao Y., Khan N.A.K., Abdullah A.L.B., Shau-Hwai A.T., Li Y.M. (2024). Integrin A5B1-mediated endocytosis of polystyrene nanoplastics: Implications for human lung disease and therapeutic targets. Sci. Total Environ..

[23] Oberländer J., Champanhac C., da Costa Marques R., Landfester K., Mailänder V. (2022). Temperature, concentration, and surface modification influence the cellular uptake and the protein corona of polystyrene nanoparticles. Acta Biomater..

[24] Zhu J., Wang J., Chen R., Feng Q., Zhan X. (2022). Cellular Process of Polystyrene Nanoparticles Entry into Wheat Roots. Environ. Sci. Technol..

[25] Xu X., Feng Y., Han C., Yao Z., Liu Y., Luo C., Sheng J. (2023). Autophagic response of intestinal epithelial cells exposed to polystyrene nanoplastics. Environ. Toxicol..

[26] Chen Y., Hua Y., Li X., Arslan I.M., Zhang W., Meng G. (2020). Distinct Types of Cell Death and the Implication in Diabetic Cardiomyopathy. Front. Pharmacol..

[27] Wang W., Zhou C., Ma Z., Zeng L., Wang H., Cheng X., Zhang C., Xue Y., Yuan Y., Li J. (2024). Co-exposure to polystyrene nanoplastics and triclosan induces synergistic cytotoxicity in human KGN granulosa cells by promoting reactive oxygen species accumulation. Ecotoxicol. Environ. Saf..

[28] Liu J., Xu F., Guo M., Gao D., Song Y. (2024). Nasal instillation of polystyrene nanoplastics induce lung injury via mitochondrial DNA release and activation of the cyclic GMP-AMP synthase-stimulator of interferon genes-signaling cascade. Sci. Total Environ..

[29] Wu Q., Liu C., Liu D., Wang Y., Qi H., Liu X., Zhang Y., Chen H., Zeng Y., Li J. (2024). Polystyrene nanoplastics-induced lung apoptosis and ferroptosis via ROS-dependent endoplasmic reticulum stress. Sci. Total Environ..

[30] Ding R., Chen Y., Shi X., Li Y., Yu Y., Sun Z., Duan J. (2024). Size-dependent toxicity of polystyrene microplastics on the gastrointestinal tract: Oxidative stress related-DNA damage and potential carcinogenicity. Sci. Total Environ..

[31] Contino M., Ferruggia G., Indelicato S., Pecoraro R., Scalisi E.M., Bracchitta G., Dragotto J., Salvaggio A., Brundo M.V. (2023). In Vitro Nano-Polystyrene Toxicity: Metabolic Dysfunctions and Cytoprotective Responses of Human Spermatozoa. Biology.

[32] Liang Y., Yang Y., Lu C., Cheng Y., Jiang X., Yang B., Li Y., Chen Q., Ao L., Cao J. (2024). Polystyrene nanoplastics exposure triggers spermatogenic cell senescence via the Sirt1/ROS axis. Ecotoxicol. Environ. Saf..

[33] Zhang M., Shi J., Pan H., Zhu J., Wang X., Song L., Deng H. (2024). A novel tiRNA-Glu-CTC induces nanoplastics accelerated vascular smooth muscle cell phenotypic switching and vascular injury through mitochondrial damage. Sci. Total Environ..

[34] Zhong G., Qiao B., He Y., Liu H., Hong P., Rao G., Tang L., Tang Z., Hu L. (2024). Co-exposure of arsenic and polystyrene-nanoplastics induced kidney injury by disrupting mitochondrial homeostasis and mtROS-mediated ferritinophagy and ferroptosis. Pestic. Biochem. Physiol..

[35] Qiu W., Ye J., Su Y., Zhang X., Pang X., Liao J., Wang R., Zhao C., Zhang H., Hu L. (2023). Co-exposure to environmentally relevant concentrations of cadmium and polystyrene nanoplastics induced oxidative stress, ferroptosis and excessive mitophagy in mice kidney. Environ. Pollut..

[36] Yang S., Zhang T., Ge Y., Cheng Y., Yin L., Pu Y., Chen Z., Liang G. (2023). Ferritinophagy Mediated by Oxidative Stress-Driven Mitochondrial Damage Is Involved in the Polystyrene Nanoparticles-Induced Ferroptosis of Lung Injury. ACS Nano.

[37] Zhang C., Li Y., Yu H., Ye L., Li T., Zhang X., Wang C., Li P., Ji H., Gao Q. (2023). Nanoplastics promote arsenic-induced ROS accumulation, mitochondrial damage and disturbances in neurotransmitter metabolism of zebrafish (Danio rerio). Sci. Total Environ..

[38] Zhang Y., Jia Z., Gao X., Zhao J., Zhang H. (2024). Polystyrene nanoparticles induced mammalian intestine damage caused by blockage of BNIP3/NIX-mediated mitophagy and gut microbiota alteration. Sci. Total Environ..

[39] Kantha P., Liu S.T., Horng J.L., Lin L.Y. (2022). Acute exposure to polystyrene nanoplastics impairs skin cells and ion regulation in zebrafish embryos. Aquat. Toxicol..

[40] Xu D., Ma Y., Peng C., Gan Y., Wang Y., Chen Z., Han X., Chen Y. (2023). Differently surface-labeled polystyrene nanoplastics at an environmentally relevant concentration induced Crohn’s ileitis-like features via triggering intestinal epithelial cell necroptosis. Environ. Int..

[41] Huang Y., Liang B., Li Z., Zhong Y., Wang B., Zhang B., Du J., Ye R., Xian H., Min W. (2023). Polystyrene nanoplastic exposure induces excessive mitophagy by activating AMPK/ULK1 pathway in differentiated SH-SY5Y cells and dopaminergic neurons in vivo. Part. Fibre Toxicol..

[42] Barguilla I., Domenech J., Rubio L., Marcos R., Hernández A. (2022). Nanoplastics and Arsenic Co-Exposures Exacerbate Oncogenic Biomarkers under an In Vitro Long-Term Exposure Scenario. Int. J. Mol. Sci..

[43] Shen F., Li D., Guo J., Chen J. (2022). Mechanistic toxicity assessment of differently sized and charged polystyrene nanoparticles based on human placental cells. Water Res..

[44] Martin-Folgar R., González-Caballero M.C., Torres-Ruiz M., Cañas-Portilla A.I., de Alba González M., Liste I., Morales M. (2024). Molecular effects of polystyrene nanoplastics on human neural stem cells. PLoS ONE.

[45] He Y., Yu T., Li H., Sun Q., Chen M., Lin Y., Dai J., Wang W., Li Q., Ju S. (2024). Polystyrene nanoplastic exposure actives ferroptosis by oxidative stress-induced lipid peroxidation in porcine oocytes during maturation. J. Anim. Sci. Biotechnol..

[46] Ge Y., Yang S., Zhang T., Gong S., Wan X., Zhu Y., Fang Y., Hu C., Yang F., Yin L. (2024). Ferroptosis participated in inhaled polystyrene nanoplastics-induced liver injury and fibrosis. Sci. Total Environ..

[47] Wen Y., Deng S., Wang B., Zhang F., Luo T., Kuang H., Kuang X., Yuan Y., Huang J., Zhang D. (2024). Exposure to polystyrene nanoplastics induces hepatotoxicity involving NRF2-NLRP3 signaling pathway in mice. Ecotoxicol. Environ. Saf..

[48] Li Z., Huang Y., Zhong Y., Liang B., Yang X., Wang Q., Sui H., Huang Z. (2023). Impact of food matrices on the characteristics and cellular toxicities of ingested nanoplastics in a simulated digestive tract. Food Chem. Toxicol..

[49] Zhao M., Xie J., Zhang J., Zhao B., Zhang Y., Xue J., Zhang R., Zhang R., Wang H., Li Y. (2024). Disturbance of mitochondrial dynamics led to spermatogenesis disorder in mice exposed to polystyrene micro- and nanoplastics. Environ. Pollut..

[50] Weber A., Schwiebs A., Solhaug H., Stenvik J., Nilsen A.M., Wagner M., Relja B., Radeke H.H. (2022). Nanoplastics affect the inflammatory cytokine release by primary human monocytes and dendritic cells. Environ. Int..

[51] Huang J., Sun X., Wang Y., Su J., Li G., Wang X., Yang Y., Zhang Y., Li B., Zhang G. (2023). Biological interactions of polystyrene nanoplastics: Their cytotoxic and immunotoxic effects on the hepatic and enteric systems. Ecotoxicol. Environ. Saf..

[52] Han S.W., Choi J., Ryu K.Y. (2021). Stress Response of Mouse Embryonic Fibroblasts Exposed to Polystyrene Nanoplastics. Int. J. Mol. Sci..

[53] Annangi B., Villacorta A., López-Mesas M., Fuentes-Cebrian V., Marcos R., Hernández A. (2023). Hazard Assessment of Polystyrene Nanoplastics in Primary Human Nasal Epithelial Cells, Focusing on the Autophagic Effects. Biomolecules.

[54] Fan Z., Zhang Y., Fang Y., Zhong H., Wei T., Akhtar H., Zhang J., Yang M., Li Y., Zhou X. (2024). Polystyrene nanoplastics induce lipophagy via the AMPK/ULK1 pathway and block lipophagic flux leading to lipid accumulation in hepatocytes. J. Hazard. Mater..

[55] Lu Y.Y., Lu L., Ren H.Y., Hua W., Zheng N., Huang F.Y., Wang J., Tian M., Huang Q. (2024). The size-dependence and reversibility of polystyrene nanoplastics-induced lipid accumulation in mice: Possible roles of lysosomes. Environ. Int..

[56] Li S., Ma Y., Ye S., Su Y., Hu D., Xiao F. (2022). Endogenous hydrogen sulfide counteracts polystyrene nanoplastics-induced mitochondrial apoptosis and excessive autophagy via regulating Nrf2 and PGC-1α signaling pathway in mouse spermatocyte-derived GC-2spd(ts) cells. Food Chem. Toxicol..

[57] Park Y.H., Jeong S.H., Yi S.M., Choi B.H., Kim Y.R., Kim I.K., Kim M.K., Son S.W. (2011). Analysis for the potential of polystyrene and TiO_2_ nanoparticles to induce skin irritation, phototoxicity, and sensitization. Toxicol. In Vitro.

[58] Dorney J., Bonnier F., Garcia A., Casey A., Chambers G., Byrne H.J. (2012). Identifying and localizing intracellular nanoparticles using Raman spectroscopy. Analyst.

[59] Xue Y., Cheng X., Ma Z.Q., Wang H.P., Zhou C., Li J., Zhang D.L., Hu L.L., Cui Y.F., Huang J. (2024). Polystyrene nanoplastics induce apoptosis, autophagy, and steroidogenesis disruption in granulosa cells to reduce oocyte quality and fertility by inhibiting the PI3K/AKT pathway in female mice. J. Nanobiotechnol..

[60] Chen Y., Liu Y., Li Y., Yao C., Qu J., Tang J., Chen G., Han Y. (2024). Acute exposure to polystyrene nanoplastics induces unfolded protein response and global protein ubiquitination in lungs of mice. Ecotoxicol. Environ. Saf..

[61] Chen J., Xu Z., Liu Y., Mei A., Wang X., Shi Q. (2023). Cellular absorption of polystyrene nanoplastics with different surface functionalization and the toxicity to RAW264.7 macrophage cells. Ecotoxicol. Environ. Saf..

[62] Lu Y.Y., Li H., Ren H., Zhang X., Huang F., Zhang D., Huang Q., Zhang X. (2022). Size-dependent effects of polystyrene nanoplastics on autophagy response in human umbilical vein endothelial cells. J. Hazard. Mater..

[63] Wang J., Zhu J., Zheng Q., Wang D., Wang H., He Y., Wang J., Zhan X. (2023). In vitro wheat protoplast cytotoxicity of polystyrene nanoplastics. Sci. Total Environ..

[64] Florance I., Chandrasekaran N., Gopinath P.M., Mukherjee A. (2022). Exposure to polystyrene nanoplastics impairs lipid metabolism in human and murine macrophages in vitro. Ecotoxicol. Environ. Saf..

[65] Yan L., Yu Z., Lin P., Qiu S., He L., Wu Z., Ma L., Gu Y., He L., Dai Z. (2023). Polystyrene nanoplastics promote the apoptosis in Caco-2 cells induced by okadaic acid more than microplastics. Ecotoxicol. Environ. Saf..

[66] Li Y., Guo M., Niu S., Shang M., Chang X., Sun Z., Zhang R., Shen X., Xue Y. (2023). ROS and DRP1 interactions accelerate the mitochondrial injury induced by polystyrene nanoplastics in human liver HepG2 cells. Chem. Biol. Interact..

[67] Hu Y., Jiang S., Zhang Q., Zhou W., Liang J., Xu Y., Su W. (2024). Protective effect of Cordycepin on blood-testis barrier against pre-puberty polystyrene nanoplastics exposure in male rats. Part. Fibre Toxicol..

[68] Xuan L., Wang Y., Qu C., Yi W., Yang J., Pan H., Zhang J., Chen C., Bai C., Zhou P.K. (2024). Exposure to polystyrene nanoplastics induces abnormal activation of innate immunity via the cGAS-STING pathway. Ecotoxicol. Environ. Saf..

[69] Mao Z., Zhong K., Liu X., Zeng X. (2023). Ferroptosis contributes to cyclophosphamide-induced hemorrhagic cystitis. Chem. Biol. Interact..

[70] Liu L., Luo C., Zheng D., Wang X., Wang R., Ding W., Shen Z., Xue P., Yu S., Liu Y. (2024). TRPML1 contributes to antimony-induced nephrotoxicity by initiating ferroptosis via chaperone-mediated autophagy. Food Chem. Toxicol..

[71] Wu Y., Wang J., Zhao T., Sun M., Xu M., Che S., Pan Z., Wu C., Shen L. (2024). Polystyrenenanoplastics lead to ferroptosis in the lungs. J. Adv. Res..

[72] Li C., Chen X., Du Z., Geng X., Li M., Yang X., Bo C., Jia Q., Yu G., Shi L. (2024). Inhibiting ferroptosis in brain microvascular endothelial cells: A potential strategy to mitigate polystyrene nanoplastics–induced blood–brain barrier dysfunction. Environ. Res..

[73] Fu X., Han H., Yang H., Xu B., Dai W., Liu L., He T., Du X., Pei X. (2024). Nrf2-mediated ferroptosis of spermatogenic cells involved in male reproductive toxicity induced by polystyrene nanoplastics in mice. J. Zhejiang Univ. Sci. B.

[74] Shi B., Xu T., Chen T., Xu S., Yao Y. (2024). Co-exposure of decabromodiphenyl ethane and polystyrene nanoplastics damages grass carp (Ctenopharyngodon idella) hepatocytes: Focus on the role of oxidative stress, ferroptosis, and inflammatory reaction. Sci. Total Environ..

[75] Chen J., Yan L., Zhang Y., Liu X., Wei Y., Zhao Y., Li K., Shi Y., Liu H., Lai W. (2024). Maternal exposure to nanopolystyrene induces neurotoxicity in offspring through P53-mediated ferritinophagy and ferroptosis in the rat hippocampus. J. Nanobiotechnol..

[76] Lu Y.Y., Hua W., Lu L., Tian M., Huang Q. (2024). The size-dependence and reversibility of polystyrene nanoplastics-induced hepatic pyroptosis in mice through TXNIP/NLRP3/GSDMD pathway. Toxicol. Res..

[77] Qin Y., Gu T., Ling J., Luo J., Zhao J., Hu B., Hua L., Wan C., Jiang S. (2022). PFOS facilitates liver inflammation and steatosis: An involvement of NLRP3 inflammasome-mediated hepatocyte pyroptosis. J. Appl. Toxicol..

[78] Wu Y., Yao Y., Bai H., Shimizu K., Li R., Zhang C. (2023). Investigation of pulmonary toxicity evaluation on mice exposed to polystyrene nanoplastics: The potential protective role of the antioxidant N-acetylcysteine. Sci. Total Environ..

[79] Chen X., Xuan Y., Chen Y., Yang F., Zhu M., Xu J., Chen J. (2024). Polystyrene nanoplastics induce intestinal and hepatic inflammation through activation of NF-κB/NLRP3 pathways and related gut-liver axis in mice. Sci. Total Environ..

[80] Ye J., Qiu W., Pang X., Su Y., Zhang X., Huang J., Xie H., Liao J., Tang Z., Chen Z. (2024). Polystyrene nanoplastics and cadmium co-exposure aggravated cardiomyocyte damage in mice by regulating PANoptosis pathway. Environ. Pollut..

[81] Halimu G., Zhang Q., Liu L., Zhang Z., Wang X., Gu W., Zhang B., Dai Y., Zhang H., Zhang C. (2022). Toxic effects of nanoplastics with different sizes and surface charges on epithelial-to-mesenchymal transition in A549 cells and the potential toxicological mechanism. J. Hazard. Mater..

[82] Zhang L., Qin Z., Bai H., Xue M., Tang J. (2024). Photochemically induced aging of polystyrene nanoplastics and its impact on norfloxacin adsorption behavior. Sci. Total Environ..

[83] Li Y., Li Y., Li J., Song Z., Zhang C., Guan B. (2023). Toxicity of polystyrene nanoplastics to human embryonic kidney cells and human normal liver cells: Effect of particle size and Pb^2+^ enrichment. Chemosphere.

[84] Chen J., Chen X., Xuan Y., Shen H., Tang Y., Zhang T., Xu J. (2023). Surface functionalization-dependent inflammatory potential of polystyrene nanoplastics through the activation of MAPK/ NF-κB signaling pathways in macrophage Raw 264.7. Ecotoxicol. Environ. Saf..

[85] Xiao S., Wang J., Digiacomo L., Amici A., De Lorenzi V., Pugliese L.A., Cardarelli F., Cerrato A., Laganà A., Cui L. (2024). Protein corona alleviates adverse biological effects of nanoplastics in breast cancer cells. Nanoscale.

[86] Domenech J., de Britto M., Velázquez A., Pastor S., Hernández A., Marcos R., Cortés C. (2021). Long-Term Effects of Polystyrene Nanoplastics in Human Intestinal Caco-2 Cells. Biomolecules.

[87] des Rieux A., Fievez V., Théate I., Mast J., Préat V., Schneider Y.J. (2007). An improved in vitro model of human intestinal follicle-associated epithelium to study nanoparticle transport by M cells. Eur. J. Pharm. Sci..

[88] Esch M.B., Mahler G.J., Stokol T., Shuler M.L. (2014). Body-on-a-chip simulation with gastrointestinal tract and liver tissues suggests that ingested nanoparticles have the potential to cause liver injury. Lab Chip.

[89] Walczak A.P., Kramer E., Hendriksen P.J., Helsdingen R., van der Zande M., Rietjens I.M., Bouwmeester H. (2015). In vitro gastrointestinal digestion increases the translocation of polystyrene nanoparticles in an in vitro intestinal co-culture model. Nanotoxicology.

[90] Fröhlich E., Bonstingl G., Höfler A., Meindl C., Leitinger G., Pieber T.R., Roblegg E. (2013). Comparison of two in vitro systems to assess cellular effects of nanoparticles-containing aerosols. Toxicol. In Vitro.

[91] Meziu E., Shehu K., Koch M., Schneider M., Kraegeloh A. (2023). Impact of mucus modulation by N-acetylcysteine on nanoparticle toxicity. Int. J. Pharm. X.

[92] Shruti V.C., Pérez-Guevara F., Elizalde-Martínez I., Kutralam-Muniasamy G. (2021). Toward a unified framework for investigating micro(nano)plastics in packaged beverages intended for human consumption. Environ. Pollut..

[93] Cella C., La Spina R., Mehn D., Fumagalli F., Ceccone G., Valsesia A., Gilliland D. (2022). Detecting Micro- and Nanoplastics Released from Food Packaging: Challenges and Analytical Strategies. Polymers.

[94] Jung S., Raghavendra A.J., Patri A.K. (2023). Comprehensive analysis of common polymers using hyphenated TGA-FTIR-GC/MS and Raman spectroscopy towards a database for micro- and nanoplastics identification, characterization, and quantitation. NanoImpact.

